# Establishing and validating models integrated with hematological biomarkers and clinical characteristics for the prognosis of non-esophageal squamous cell carcinoma patients

**DOI:** 10.1080/07853890.2025.2483985

**Published:** 2025-03-28

**Authors:** Ning Xue, Xiaoyan Wen, Qian Wang, Yong Shen, Yuanye Qu, Qingxia Xu, Shulin Chen, Jing Chen

**Affiliations:** a Department of Clinical Laboratory, The Affiliated Cancer Hospital of Zhengzhou University and Henan Cancer Hospital, Zhengzhou Key Laboratory of Digestive System Tumor Marker Diagnosis, Zhengzhou, P. R. China; b Central Sterilization Supply Department, The Guanghua Stomatological College of Sun Yat-sen University, Hospital of Stomatology, SunYat-sen University, Guangzhou, P. R. China; c Department of radiation oncology, China–Japan Union Hospital of Jilin University, Changchun, P.R. China; d State Key Laboratory of Oncology in South China, Guangdong Provincial Clinical Research Center for Cancer, Collaborative Innovation Center for Cancer Medicine, Sun Yat-Sen University Cancer Center, Guangzhou, China; e Research Center for Translational Medicine, First Affiliated Hospital of Sun Yat-Sen University, Guangzhou, P. R. China

**Keywords:** LASSO Cox model, non-esophageal squamous cell carcinoma, prognostic model, RSF model

## Abstract

**Background:**

This study aimed to construct a novel model and validate its predictive power in non-esophageal squamous cell carcinoma (NESCC) patients.

**Methods:**

This retrospective study included 151 patients between October 2006 and September 2016. The LASSO Cox and Random Survival Forest (RSF) models were developed with the help of hematological biomarkers and clinical characteristics. The concordance index (C-index) was used to assess the prognostic power of the LASSO Cox model, RSF model, and TNM staging. Based on the risk scores of the LASSO Cox and RSF models, we divided patients into low-risk and high-risk subgroups.

**Results:**

We constructed two models in NESCC patients according to LASSO Cox regression and RSF models. The RSF model reached a C-index of 0.841 (95% CI: 0.792–0.889) in the primary cohort and 0.880 (95% CI: 0.830–0.930) in the validation cohort, which was higher than the C-index of the LASSO Cox model 0.656 (95% CI: 0.580–0.732) and 0.632 (95% CI: 0.542–0.720) in the two cohorts. The integrated C/D area under the ROC curve (AUC) values for the LASSO Cox and RSF models were 0.701 and 0.861, respectively. In both two models, Kaplan-Meier survival analysis and the estimated restricted mean survival time (RMST) values indicated that the low-risk subgroup had a better prognostic outcome than the high-risk subgroup (*p* < 0.05).

**Conclusions:**

The RSF model has better prediction power than the LASSO Cox and the TNM staging models. It has a guiding value for the choice of individualized treatment in patients with NESCC.

## Introduction

Esophageal cancer (EC) is one of the leading causes of tumor-induced morbidity and mortality worldwide [1,2]. The most common histological subtype of EC is esophageal squamous cell carcinoma (ESCC), which also includes adenocarcinoma, adenosquamous carcinoma, neuroendocrine carcinoma, and esophageal sarcomatoid carcinoma, among others., and non-esophageal squamous cell carcinoma (NESCC) accounts for approximately 10% of all ECs [3]. In developing countries including China, NESCC is rare, and more than 90% patients with EC are diagnosed with ESCC [4]. However, esophageal adenocarcinoma represents 80% of all cases of ECs in United States [5]. EC is usually diagnosed at a later stage, with high mortality and a 5-year overall survival (OS) rate of 15–20% [6]. One previous study showed that patients with ESCC had better OS outcomes than patients with adenocarcinoma of the esophagus after the same treatment [7]. Many studies have focused on the prognosis of ESCC. However, only few studies have focused on the prognosis of NESCC. Thus, it is indispensable to implement a novel model for the prognosis of patients with NESCC, which will then allow the patients to receive more precise and individualized treatment.

With the popularity of electronic medical records and improvements in laboratory examinations, numerous prognostic models have been developed and widely applied in clinical practice. A clinical prognostic model based on albumin-to-fibrinogen ratio (AFR) and neutrophil–lymphocyte ratio (NLR) can be used to predict the prognosis of ESCC patients after radical resection [8]. Luo et al. established a novel model based on serum IgA, CRP, and cTNM stage to evaluate the prognosis of ESCC patients receiving neoadjuvant chemoradiotherapy plus surgery [9]. Further, Wang et al. constructed a nomogram that included five variables: age, N stage, location, tumor response, and monocyte/lymphocyte ratio [10]. This nomogram can serve as an effective tool for patients with locally advanced ESCC who are undergoing concurrent chemoradiotherapy. Furthermore, a nomogram based on hemoglobin (HB), C-reactive protein-to-albumin ratio (CAR), and platelet-to-lymphocyte ratio (PLR) may accurately and effectively predict OS in ESCC [11]. However, all existing models have only been used to evaluate the prognosis of patients with ESCC.

Random survival forest (RSF) is an ensemble tree method, which is used to analyze right-censored survival data [12,13]. It adopts iterating the missing data algorithm, and its accuracy can be substantially improved even with missing data [13]. Moreover, RSF can easily be applied to real data settings to reveal highly complex interrelationships between variables [14]. In this study, RSF and LASSO Cox regression were performed to screen for the potential factors affecting the prognosis of patients with NESCC and to construct novel prediction models. We further compared the predictive power of the two prognostic models with regard to OS in compared with the 8th edition of the Union for International Cancer Control (UICC)/American Joint Committee on Cancer (AJCC) TNM staging system.

## Materials and methods

### Participants

This study is a retrospective analysis of patients with NESCC who were undergoing treatment at the Sun-Yat Sen University Cancer Center. We constructed the LASSO Cox and RSF models based on patients clinical factors. Subsequently, we compared the prediction ability of LASSO Cox, RSF, and the TNM staging models. In total, 151 patients with NESCC were enrolled in this study. All patients were treated from October 2006 to September 2016 at the Sun-Yat Sen University Cancer Center. The inclusion criteria were as follows: (a) NESCC diagnosed by two independent pathologists, (b) absence of any second carcinoma, (c) no antitumor therapy or anti-inflammatory treatment, (d) complete clinical and laboratory findings. The patients were followed up every 3 months. The last follow-up was conducted in March 2024. The follow-up end-point was cancer-specific death or March 2024. Follow-up data were obtained from medical records, clinics, and telephone inquiries. The TNM stage was based on the 8th edition of the Union for International Cancer Control (UICC)/American Joint Committee on Cancer (AJCC) TNM staging system.

This study was approved by the Clinical Research Ethics Committee of Sun Yat-Sen University Cancer Center and was conducted in accordance with the Declaration of Helsinki. Given that this is a retrospective study, the Clinical Research Ethics Committee of Sun Yat-sen University Cancer Center waived the need for informed consent.

### Clinical data collection

We collected the following clinical data for each patient: Age, Gender, location, Histological type, white blood cell (WBC) count, neutrophil (NEU) count, lymphocyte (LYM) count, monocyte (MO) count, hemoglobin (HGB), platelet count (PLT), C-reactive protein (CRP), body mass index (BMI), Glucose(GLU), the advanced lung cancer inflammation index (ALI), nutritional risk index (NRI), NLR, LYM/MO ratio (LMR), PLT/LYM ratio (PLR), NEU/LYM ratio (NLR), LYM/CRP ratio (LCR), GLU/LYM ratio (GLR), prognostic nutritional index (PNI), alanine aminotransferase (ALT), aspartate aminotransferase (AST), ALT/AST ratio (LSR), alkaline phosphatase (ALP), glutamyl transpeptidase (GGT), lactic dehydrogenase (LDH), LDH/CRP ratio (LDCR), albumin (ALB), CRP/ALB ratio (CAR), total protein (TP), globulin (GLOB), ALB/GLOB ratio (AGR), total bilirubin (TBIL), direct bilirubin (DBIL), blood urea nitrogen (BUN), creatinine (CRE), uric acid (UA), cystatin C (CYSC), triglyceride (TG), cholesterol (CHO), high density lipoprotein (HDL), TG/HDL ratio (THR), low density lipoprotein (LDL), LDL/HDL ratio (LHR), apolipoprotein A (APOA), apolipoprotein B (APOB), APOA/APOB ratio (ABR), systemic immune-inflammation index (SII), systemic inflammation response index (SIRI), aggregate index of systemic inflammation (AISI), and prognostic impact index. The relative calculation formulae were as follows: SII = NEU × PLT/LYM [15]. NRI = 1.487 × ALB (g/L) + 41.7 × preoperative weight/ideal body weight (kg) [16]. Ideal body weight = 22 × height (m)^2^. PNI = ALB (g/L) + 5 × LYM × 10^9^/L [17]. SIRI = NEU × MO/LYM. AISI = NEU × MO × PLT/LYM [18].

### Statistical analysis

All statistical analyses were performed using the R software (version 3.6.2) and SPSS 25.0 (SPSS, Chicago, USA). LASSO Cox regression (‘glmnet’ R package) and RSF (‘randomForestSRC’ R package) were used to construct models in the primary cohort, and potential factors were screened through cross-validation to determine the final factors. LASSO Cox model used L1 regularization to reduce the regression coefficients toward zero, and reduce the risk of overfitting. The RSF algorithm can measure the importance of factors, and rank factors according to their importance. Performance evaluation for the LASSO Cox model, RSF model, and TNM staging model was performed using the integrated Brier score (IBS), concordance index (C-index), and receiver operating characteristic curve (ROC). The C-index and area under the ROC curve (AUC) were calculated using the R package ‘survcomp’ and R package ‘survivalROC’. Based on the cutoff values (‘survminer’ R package), patients were divided into low-risk and high-risk subgroups in the LASSO Cox, RSF, and TNM staging models. The OS in the risk subgroups was evaluated via the Kaplan–Meier method. Data are shown as the mean ± SD. Statistical tests were two-sided, and significant differences were considered at *p* < 0.05.

## Results

### Patient characteristics

A total of 151 patients with NESCC were randomly divided into the primary cohort (*n* = 90) and the validation cohort (*n* = 61). The clinical characteristics of the primary and validation cohorts are shown in Table 1. There were 90 patients in the primary cohort, comprising 75 (83.33%) males and 15 (16.67%) females. The mean age (SD) of the patients was 58.44 (10.22) years. There were 36 (40.00%) and 54 (60.00%) I/II and III/IV stage patients, respectively. The 1-, 3-, and 5-year OS rates were 65.56%, 30.00%, and 22.22%, respectively. In the validation cohort, there were 47 (77.05%) males and 14 (22.95%) females. The mean age (SD) of these patients was 60.26 (9.28) years. There were 35 (57.38%) and 26 (42.62%) I/II and III/IV stage patients, respectively. The 1-, 3-, and 5-year OS rates in this cohort were 72.13%, 34.43%, and 27.87%, respectively.

**Table 1.** Demographics and clinical characteristics of NESCC patients in the primary and validation cohorts.

Characteristic	Primary cohort	Validation cohort	
	*n* = (90)	*n* = (61)	*p* value
	No. (%) or mean ± SD	No. (%) or mean ± SD	
Gender			0.336^a^
Female	15 (16.67%)	14 (22.95%)	
Male	75 (83.33%)	47 (77.05%)	
Age (years)	58.44 ± 10.22	60.26 ± 9.28	0.256^b^
Histologic type			0.861^a^
Adenocarcinoma	11 (12.22%)	6 (9.84%)	
Adenosquamous carcinoma	12 (13.33%)	11 (18.03%)	
Neuroendocrine carcinoma	17 (18.89%)	9 (14.75%)	
Esophageal sarcomatoid carcinoma	41 (45.56%)	30 (8.20%)	
Other	9 (10.00%)	5 (16.67%)	
TNM stage			0.036^a^
I + II	36 (40.00%)	35 (57.38%)	
III+IV	54 (60.00%)	26 (42.62%)	
Tumor location			0.411^a^
Upper	10 (11.11%)	3 (4.92%)	
Middle	52 (57.78%)	38 (62.30%)	
Lower	28 (31.11%)	20 (32.79%)	
BMI (kg/m^2^)			0.532^a^
<18.00	12 (13.33%)	5 (8.20%)	
18.00–24.00	59 (65.56%)	40 (65.57%)	
>24.00	19 (21.11%)	16 (26.23%)	
WBC (10^9^/L)	8.26 ± 3.40	7.75 ± 2.99	0.376^b^
Neutrophils (10^9^/L)	5.63 ± 3.32	5.11 ± 2.95	0.341^b^
Lymphocyte (10^9^/L)	1.74 ± 0.73	1.93 ± 0.68	0.059^b^
Monocytes (10^9^/L)	0.61 ± 0.28	0.54 ± 0.22	0.116^b^
HGB (g/L)	129.41 ± 17.76	134.37 ± 15.99	0.098^b^
Platelet (10^9^/L)	257.74 ± 88.88	227.13 ± 71.10	0.054^b^
NRI	101.01 ± 11.22	104.03 ± 11.14	0.045^b^
NLR	4.48 ± 5.18	3.53 ± 4.70	0.129^b^
LMR	3.48 ± 2.17	4.22 ± 2.63	0.040^b^
PLR	176.13 ± 112.86	130.62 ± 58.50	0.008^b^
LCR	1.04 ± 1.63	1.81 ± 2.69	0.003^b^
GLR	4.09 ± 2.94	3.38 ± 2.12	0.061^b^
TBIL (μmol/L)	12.70 ± 9.71	11.83 ± 5.56	0.862^b^
DBIL (μmol/L)	4.46 ± 5.42	4.00 ± 2.60	0.685^b^
TP (g/L)	70.73 ± 5.86	70.72 ± 5.52	0.743^b^
ALB (g/L)	40.64 ± 6.07	41.91 ± 6.29	0.059^b^
GLB (g/L)	30.24 ± 5.96	28.17 ± 4.92	0.019^b^
AGR	1.40 ± 0.31	1.57 ± 0.38	0.020^b^
ALT (U/L)	18.84 ± 16.40	22.98 ± 29.74	0.678^b^
AST (U/L)	20.37 ± 10.37	22.67 ± 18.85	0.897^b^
LSR	0.89 ± 0.30	0.92 ± 0.30	0.422^b^
ALP (U/L)	76.67 ± 35.53	78.84 ± 37.40	0.634^b^
GGT (U/L)	40.95 ± 63.08	47.34 ± 114.34	0.675^b^
LDH (U/L)	179.80 ± 72.36	172.45 ± 73.76	0.256^b^
LDCR	111.45 ± 242.18	148.27 ± 202.48	0.008^b^
CRP (mg/L)	21.42 ± 35.72	6.79 ± 0.90	0.005^b^
CAR	2.56 ± 19.11	0.18 ± 0.33	0.006^b^
CRE (μmol/L)	76.77 ± 21.21	76.69 ± 20.67	0.958^b^
BUN (mmol/L)	9.55 ± 43.28	5.27 ± 2.12	0.844^b^
UA (μmol/L)	332.26 ± 100.79	338.52 ± 80.80	0.553^b^
CYSC (mg/L)	1.70 ± 7.03	0.93 ± 0.29	0.262^b^
GLU (mmol/L)	5.65 ± 1.29	5.49 ± 1.44	0.448^b^
TG (mmol/L)	1.22 ± 0.55	1.22 ± 0.58	0.971^b^
CHO (mmol/L)	5.06 ± 1.00	4.97 ± 1.04	0.522^b^
HDL (mmol/L)	1.12 ± 0.30	1.21 ± 0.33	0.110^b^
LDL (mmol/L)	3.71 ± 3.75	3.18 ± 0.90	0.171^b^
THR	1.23 ± 0.82	1.14 ± 0.92	0.418^b^
LHR	3.41 ± 2.58	2.78 ± 0.93	0.014^b^
APOA (g/L)	1.12 ± 0.22	1.25 ± 0.25	0.002^b^
APOB (g/L)	1.01 ± 0.21	1.03 ± 0.58	0.191^b^
ABR	1.16 ± 0.35	1.36 ± 0.56	0.007^b^
ALI	373.27 ± 272.34	460.88 ± 294.48	0.046^b^
SII	1217.86 ± 1624.63	746.22 ± 881.18	0.037^b^
AISI	980.30 ± 1824.88	449.89 ± 682.80	0.030^b^
SIRI	3.45 ± 6.01	2.25 ± 4.43	0.070^b^
PNI	49.36 ± 7.56	51.53 ± 8.12	0.018^b^

*Note*. a. Chi-squared test, b Wilcoxon test.

BMI, body mass index; WBC, white blood cell; HGB, hemoglobin; NRI, the nutritional risk index; NLR, neutrophil/lymphocyte ratio; LMR, lymphocyte/monocyte ratio; PLR, platelet/lymphocyte ratio; CRP, C-reactive protein; LCR, lymphocyte/CRP; GLR, glucose/lymphocyte; TBIL, bilirubin total; DBIL, direct bilirubin; TP, total protein; ALB, albumin; GLOB, globulin; AGR, ALB/GLOB ratio; ALT, alanine aminotransferase; AST, aspartate aminotransferase; SLR, AST/ALT ratio; ALP, alkaline phosphatase; GGT, glutamyl transpeptidase; LDH, lactic dehydrogenase; LDCR, LDH/CRP ratio; CAR, CRP/ALB; CRE, creatinine; BUN, blood urea nitrogen; UA, uric acid; CYSC, cystatin C; GLU, Glucose; TG, triglyceride; CHO, cholesterol; HDL, high density lipoprotein; LDL, low density lipoprotein; THR, TG/HDL ratio; LHR, LDL/HDL ratio; APOA, apolipoprotein AI; APOB, apolipoprotein B; ABR, APOA/APOB ratio; ALI, the advanced lung cancer inflammation index; SII, systemic immune-inflammation index; AISI, aggregate index of systemic inflammation; SIRI, systemic inflammation response index; PNI, prognostic impact index.

### Prognostic models for OS

**Figure 1.**
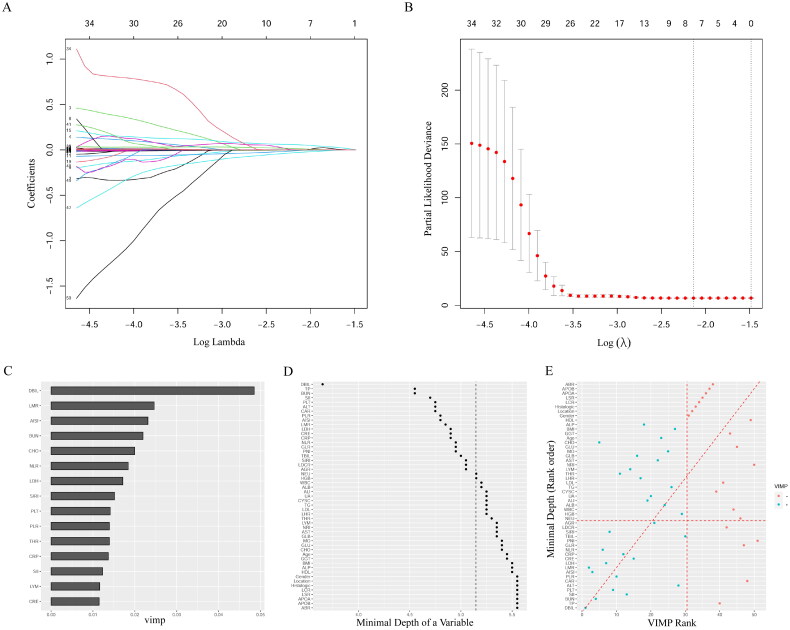
Potential features selection (A) and tenfold cross validation (B) of LASSO Cox model. Variable importance values permutation (C), minimal depth variable importance (D) and variable importance values rank (E) for patients of NESCC.

In the primary cohort, TBIL, LHR, NEU, LDCR, LMR BMI and SIRI were identified as candidate markers from 52 clinical factors by LASSO Cox regression analysis. The trajectory changes for each factor (Figure 1A) and confidence intervals for each λ (Figure 1B) are shown in Figure 1. RSF was performed to select potential factors from these clinical features. We constructed an RSF model that included DBIL, TP, BUN, PLT, and SII levels. Figure 1 shows the variable importance (VIMP) values permutation (Figure 1C), minimal depth variable importance (Figure 1D) and variable importance value rank (Figure 1E) for patients with NESCC. Variables with lower values of minimal depth variable importance are implied to be more important variables. In Figure 1E, the blue dot represents a VIMP score greater than zero and the red dot represents a VIMP score less than zero. The area above the red diagonal dotted line represents a higher rank of the VIMP score, and that below the diagonal dotted line represents the minimum depth of higher rank.

### Variable interpretation and comparison of prognostic models

Because of the complexity of the algorithms, the Survex R package was used to compare the LASSO Cox model and the RSF model and explain their global features. In both the primary and validation cohorts, The C-index, the IBS loss after permutation (Supplemental Figure 1A,C) and integrated C/D AUC loss after permutation (Supplemental Figure 1B,D) were evaluated to determine the time-dependent variable importance for the LASSO Cox model and RSF model. The IBS and integrated C/D AUCs of the LASSO Cox and RSF model were 0.141, 0.701 and 0.166, 0.861, respectively. In the primary cohort, the C-index of the RSF model was 0.841(95% CI: 0.792–0.889), which was significantly higher than that of the LASSO Cox model (0.656; 95% CI: 0.580–0.732; *p* < 0.001) and TNM staging model (0.518; 95% CI: 0.444–0.592; *p* < 0.001). For the validation cohort, the C-index values of these models (RSF model, LASSO Cox model, and TNM staging model) were 0.880 (95% CI: 0.830–0.930), 0.632 (95% CI: 0.542–0.720), and 0.655 (95% CI: 0.569–0.741), respectively (Table 2). When it comes to global interpretation, the results show that the influence of the LHR feature in the LASSO Cox model gradually increased. In the RSF model, SII and DBIL are important features. Supplemental Figure 2 shows the partial-dependence survival profiles of the LASSO Cox and RSF models. LHR was the main influencing factor in the LASSO Cox model (Supplemental Figure 2A). Further, in the RSF model, DBIL and SII were the most influential factors (Supplemental Figure 2B). The AUCs of the RSF model in the primary cohort for 1-, 3-, and 5-year OS were 0.871, 0.975, and 0.970, respectively. Meanwhile, the AUCs of the LASSO Cox model in the primary cohort for 1-, 3-, and 5-year OS were 0.664, 0.771, and 0.757, respectively. The AUCs of the TNM staging model in the primary cohort for 1-, 3-, and 5-year OS were 0.533, 0.494, and 0,478, respectively (Supplemental Figure 3). Similarly, the AUCs of the RSF model for 1-, 3-, and 5-year OS were higher than those of the LASSO Cox model and TNM staging model in the validation cohort. These results demonstrate that the prediction performance of the RSF model is superior to that of the LASSO Cox model.

**Table 2.** The C-index of OS for LASSO Cox model, RSF model and TNM staging model.

Survival prediction	C-index	95 CI%	*p*
For primary cohort			
LASSO Cox model	0.656	0.580–0.732	
RSF model	0.841	0.792–0.889	
TNM staging model	0.518	0.444–0.592	
LASSO Cox model vs TNM staging model			0.003
RSF model vs TNM staging model			<0.001
LASSO Cox model vs RSF			<0.001
For validation cohort			
LASSO Cox model	0.632	0.542–0.720	
RSF model	0.880	0.830–0.930	
TNM staging model	0.655	0.569–0.741	
LASSO Cox model vs TNM staging model			0.699
RSF model vs TNM staging model			<0.001
LASSO Cox model vs RSF			<0.001

C-index = concordance index; *p* values were calculated based on normal approximation using the rcorrp.cens function in the Hmisc package.

### Model performance at stratifying risk

**Figure 2.**
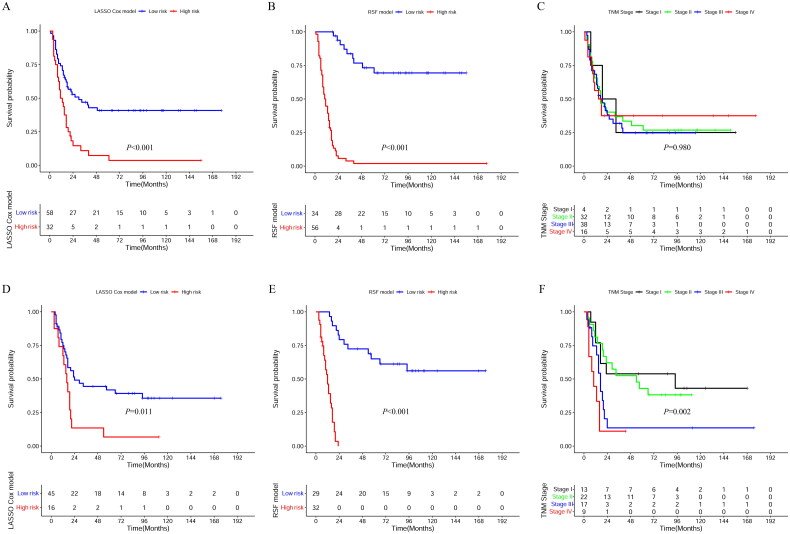
Kaplan–Meier curves of high risk and low risk subgroups for the LASSO Cox model, the RSF model, and the TNM stage in the primary cohort (A–C) and the validation cohort (D–F).

Based on the risk scores, we adopted the Survminer and Survival R package to determine cutoff values. In the LASSO Cox model, patients with a risk score less than 1.27 were in the low-risk subgroup, and the others were in the high-risk subgroup. Moreover, in the RSF model, patients with a risk score less than 15.31 were in the low-risk subgroup, and others were in the high-risk group. In the LASSO Cox model, the low-risk subgroup had longer OS than the high-risk subgroup in both the primary cohort (*p* < 0.001) (Figure 2A) and the validation cohort (*p* = 0.011) (Figure 2D). In the RSF model, the low-risk subgroup showed better OS than the high-risk subgroup in both cohorts (*p* < 0.001) (Figure 2B,E). The OS of each patient in the TNM staging group was significantly different in the validation cohort (*p* = 0.002) (Figure 2F). However, there was no significant difference in the OS between different TNM stages in the validation cohort (*p* = 0.980) (Figure 2C).

**Figure 3.**
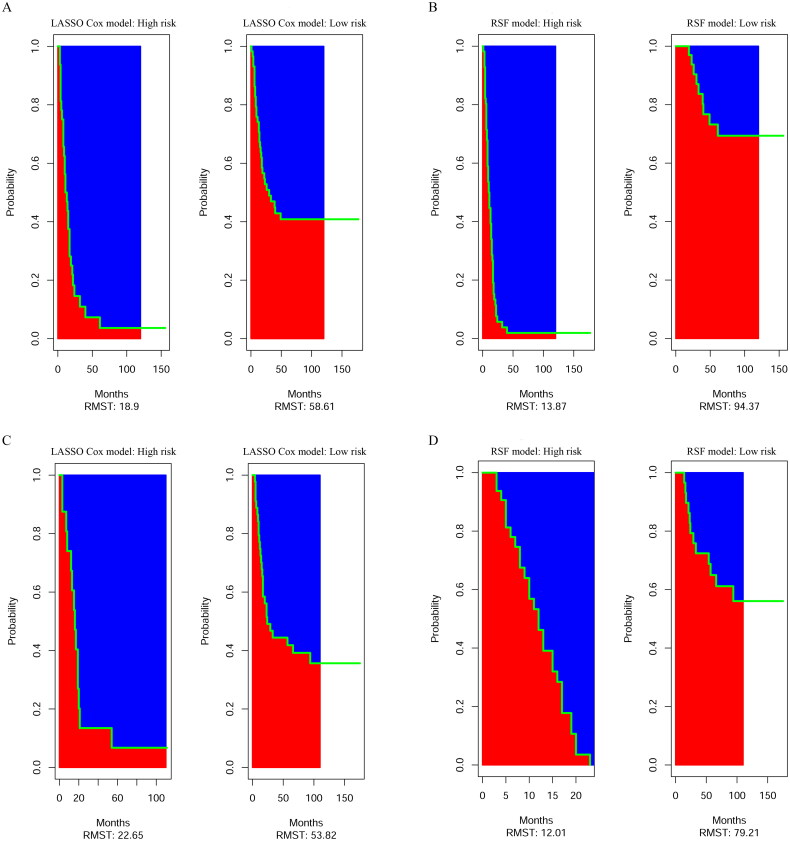
The estimate restricted mean survival time (RMST) values of the LASSO Cox model (A, C) and the RSF model in the primary cohort and the validation cohort (B, D).

**Figure 4.**
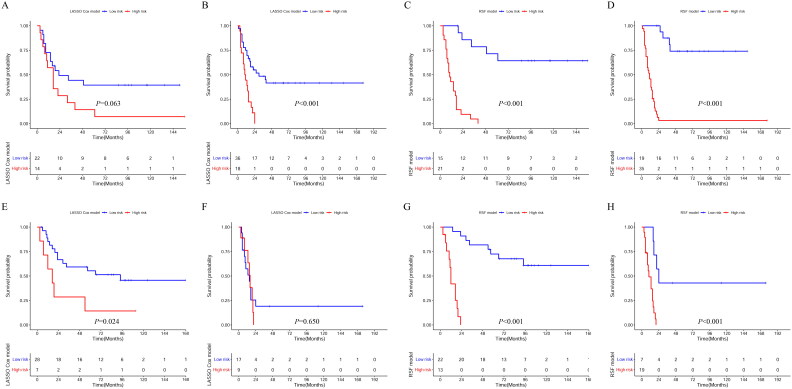
Kaplan–Meier curves of I/II and III/IV patients with NESCC in the primary and validation cohorts. LASSO Cox model: I/II patients (A) and III/IV patients (B) in the primary cohort, I/II patients (E) and III/IV patients (F) in the validation cohort; RSF model: I/II patients (C) and III/IV patients (D) in the primary cohort, I/II patients (G) and III/IV patients (H) in the validation cohort.

In the primary cohort, the estimated restricted mean survival time (RMST) values in the high-risk subgroup and low-risk subgroup in the LASSO Cox were 18.9 and 58.61 months, respectively (Figure 3A). The RMST values in the high-risk and low-risk subgroups in the RSF model were 13.87 and 94.37 months, respectively (Figure 3B). For the validation cohort, the RMST values for the high-risk and low-risk subgroups in of the LASSO Cox model and RSF model were 22.65, 53.82 months and 12.01, 79.21 months, respectively (Figure 3C,D). Meanwhile, we divided the patients into I/II and III/IV groups based on their TNM stage. In the primary cohort of the LASSO Cox model, the low-risk patients had significantly longer OS than the high-risk patients in both group I/II (*p* = 0.063) (Figure 4A) and group III/IV (*p* < 0.001) (Figure 4B). In the validation cohort of the LASSO Cox model, although the OS of low-risk and high-risk patients was significantly different in the I/II group (*p* = 0.024) (Figure 4E), there was no significant difference between the OS of low-risk and high-risk patients in stage III/IV (*p* = 0.650) (Figure 4F). In both the primary and validation cohorts, the low-risk patients showed better OS benefit than high-risk patients in both the I/II and III/IV groups in the RSF model (*p* < 0.001) (Figure 4C,D,G,H).

### Correlations among prognostic models

**Figure 5.**
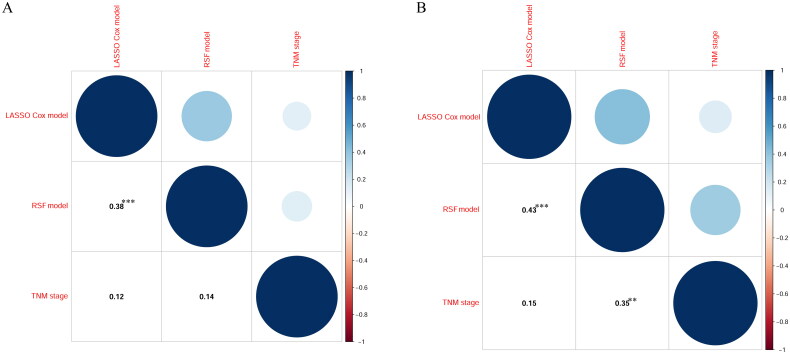
The correlations among the LASSO Cox model, RSF model, and TNM stage in the primary cohort (A) and the validation cohort (B).

Figure 5 shows the correlations among the LASSO model, RSF, and TNM staging models. The Pearson correlation coefficient (PCC) ranged from −1 to +1. Red and Blue colors represent negative and positive correlations, respectively. In the primary cohort, the LASSO Cox model weakly correlated with the RSF model [Pearson correlation coefficient (PCC): 0.38]. Meanwhile, the LASSO Cox model moderately correlated with the RSF model in the validation cohort (PCC: 0.43). Moreover, there was a weak correlation between the TNM staging model and RSF model in the validation cohort (PCC: 0.35).

## Discussion

EC with different histological types and differences in epidemiology, pathogenesis, and risk factors. Therefore, the survival times of patients with EC with different histological types are different. Most prognostic models for EC focus on ESCC, and those for NESCC are rare. In the present study, the LASSO Cox and RSF models were developed for patients with NESCC. Moreover, the LASSO Cox and RSF models were found to have better prediction performances than the traditional TNM staging system.

In this study, we constructed LASSO Cox and RSF models for patients with NESCC. The LASSO Cox model included seven factors (TBIL, LHR, NEU, LDCR, LMR BMI and SIRI) and the RSF model included five factors (DBIL, TP, BUN, PLT, and SII). The LASSO Cox model based on the LASSO Cox regression algorithm achieved a C-index of 0.656 (95% CI: 0.580–0.732), IBS of 0.166, and integrated C/D AUC of 0.701. Similarly, the RSF model using the random forest algorithm reached a C-index of 0.841 (95% CI: 0.792–0.889), IBS of 0.141, and integrated C/D AUC of 0.861. In both the primary and validation cohorts, the RSF model achieved a higher AUC than the LASSO Cox model and TNM staging model in terms of 1-, 3-, and 5-year OS of patients with NESCC. According to the respective cutoff values, NESCC patients were divided into low risk and high-risk subgroups. In both the primary and validation cohorts of the LASSO Cox model, patients in the low-risk subgroup had a longer OS than those in the high-risk subgroup. Similar results were observed in the RSF model.

To the best of our knowledge, this is the first study to construct prognostic models for NESCC based on the RSF and LASSO Cox regression. This study developed an RSF prognostic model superior to the LASSO Cox model and the traditional TNM staging system for patients with NESCC. The advantage of RSF is that it is not constrained by proportional hazards assumption, log-linear assumption and other conditions [19,20]. The prediction by RSF was at least equal to or better than that by traditional survival analysis methods such as the Cox proportional hazards regression model [21–23]. RSF can also perform survival analysis and variable screening of high-dimensional data and can be applied to the analysis of competing risks [24–26]. Moreover, RSF has been widely used for the analysis of time-to-event outcomes and the construction of clinical prediction models [27–31]. Based on interpretable genomic inputs, an RSF model was proposed and used to predict relapse/death in high-risk pediatric patients with acute lymphoblastic leukemia [32]. Zhang et al. constructed an RSF model to predict the 30-day mortality among elderly patients with sepsis. The RSF model showed better predictive ability than the traditional scoring system [33].

In this study, the RSF model included five factors (DBIL, TP, BUN, PLT, and SII). Studies have demonstrated that the levels of DBIL are associated with systemic oxidative stress (SOS), and a low SOS index is an important factor for better prognosis in patient with ESCC [34]. TP and ALB reflect the nutritional status of patients, and nutritional status significantly affects disease progression and survival in patients with EC [35]. The combination of HGB, ALB, LYM, and PLT score (HALP) reflects the status of inflammation and nutrition. A low HALP score has shown to be an independent risk factor for poor OS in patients with ESCC [36]. BUN is a nitrogenous end product of protein metabolism, and it can estimate the function of renal [37]. A previous study found that BUN decreased in patients with advanced oral cancer [38]. SII was an indicator of host inflammatory status. Evidence has shown that inflammatory responses were considered as important factors associated with clinical prognosis in cancers [39,40]. Thus, SII may be a promising predictor of cancer survival and tumor progression in EC. Moreover, elevated SII was significantly associated with deeper infiltration depth, positive lymph node metastasis, and advanced clinical stage [41].

This study has several limitations. First, we constructed multiple-models for patients with NESCC. However, we did not perform a detailed analysis on each histological type (adenocarcinoma, adenosquamous carcinoma, neuroendocrine carcinoma, esophageal sarcomatoid carcinoma, etc.). Second, the data of this study came from a single center and this was a retrospective study. Therefore, potential biases cannot be completely ruled out. Third, this study included a small number of patients with NESCC. We intend to collect data on a larger number of patients from other institutions and validate the RSF model.

In conclusion, we found that the LASSO Cox and RSF models were better than the TNM staging model for predicting OS in patients with NESCC. Moreover, the RSF model showed a superior discrimination ability compare with the LASSO Cox model. Our RSF model has guiding values for clinicians, which will benefit their choice of individualized treatment and follow-up prognosis for patients with NESCC.

## Supplementary Material

Supplemental Material

## Data Availability

The datasets analyzed during the current study are not publicly available because of patient privacy concerns, but are available from the corresponding author upon reasonable request.
