# Supplementary material for: Establishing and validating models integrated with hematological biomarkers and clinical characteristics for the prognosis of non-esophageal squamous cell carcinoma patients
Source: Ann Med. 2025 Mar 28;57(1):2483985. doi: 10.1080/07853890.2025.2483985 (PMC11956093; doi:10.1080/07853890.2025.2483985)
Supplement: Supplemental Material [file IANN_A_2483985_SM8562.zip › Suppl/Supplemental Figure caption.docx]

**Supplemental Figure** **1** Time-dependent feature importance of the LASSO Cox (A, B) model and the RSF model (C, D).

**Supplemental Figure 2** Partial dependence of each variable in the LASSO Cox model (A) and the RSF model (B).

**Supplemental Figure 3** ROC curves of the TNM stage, LASSO Cox model, and RSF model for 1-, 3-, and 5-years OS in the primary cohort (A-C) and the validation cohort (D-F).
