# Supplementary figures and images for: Establishing and validating models integrated with hematological biomarkers and clinical characteristics for the prognosis of non-esophageal squamous cell carcinoma patients
Source: Ann Med. 2025 Mar 28;57(1):2483985. doi: 10.1080/07853890.2025.2483985 (PMC11956093; doi:10.1080/07853890.2025.2483985)

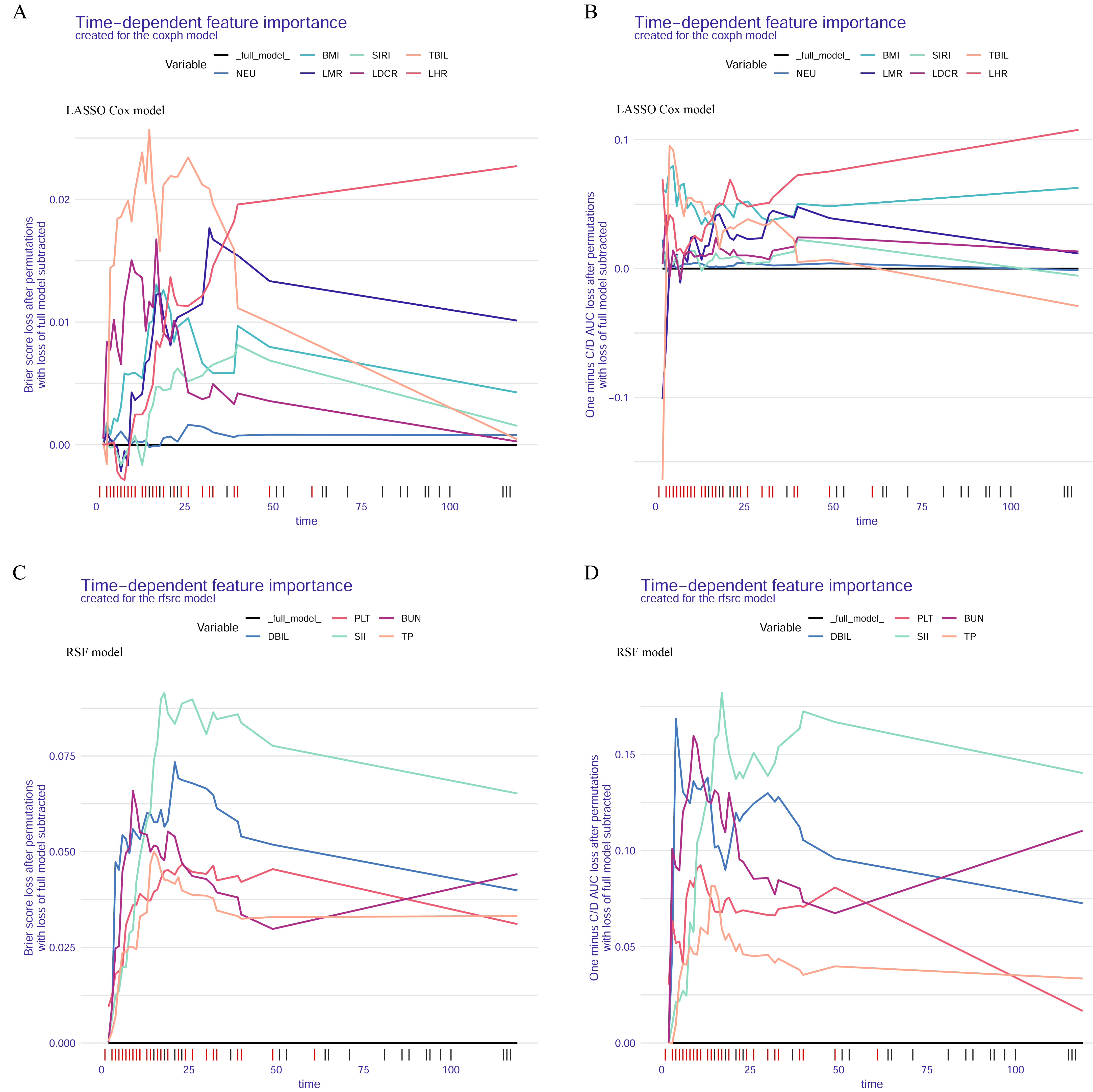

Supplement: Supplemental Material [file IANN_A_2483985_SM8562.zip › Suppl/Supplemental Figure 1.tif]

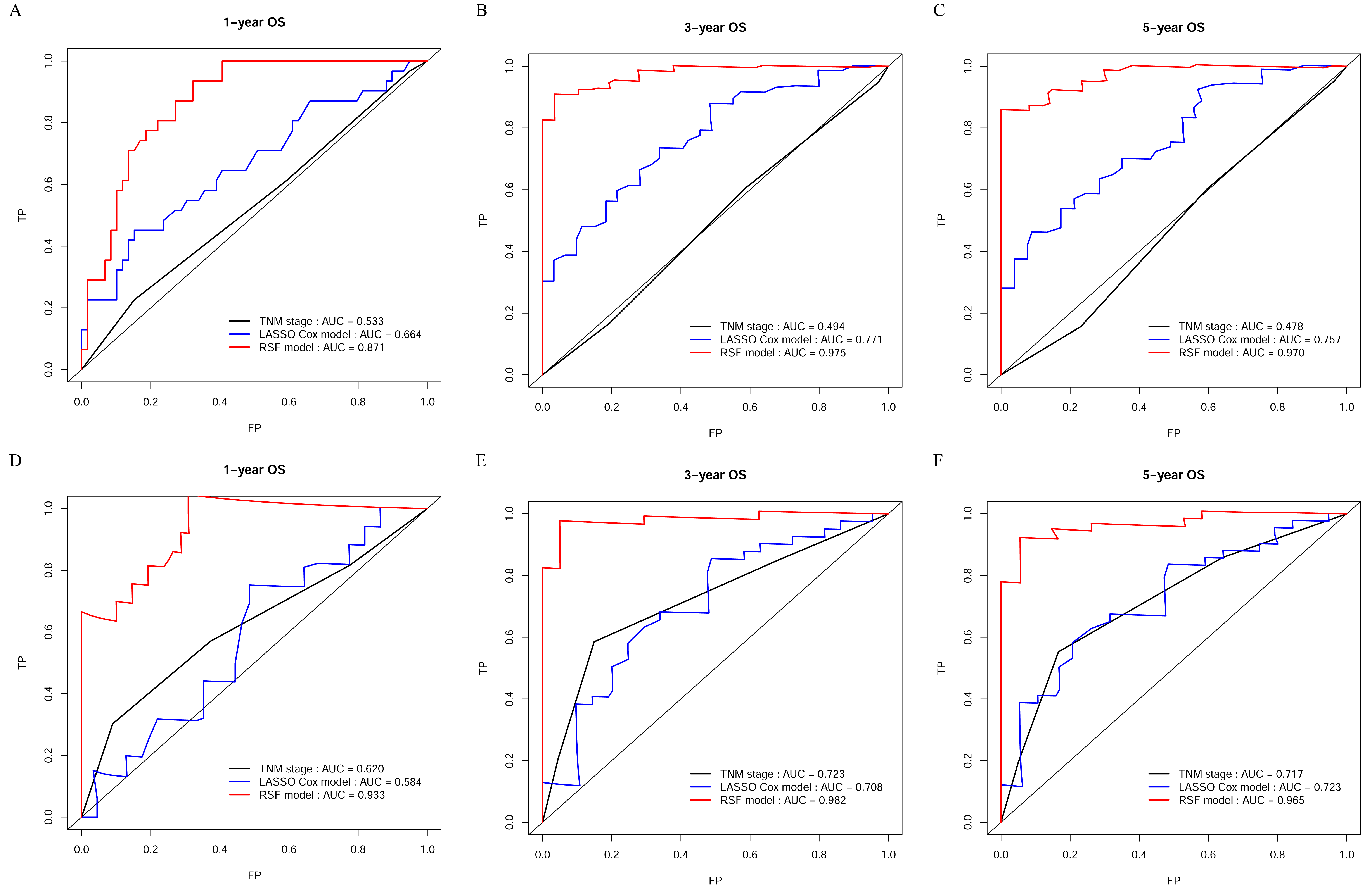

Supplement: Supplemental Material [file IANN_A_2483985_SM8562.zip › Suppl/Supplemental Figure 3.tif]
